# Measurement of Macromolecular Crowding in Rhodobacter sphaeroides under Different Growth Conditions

**DOI:** 10.1128/mbio.03672-21

**Published:** 2022-01-25

**Authors:** Jia Hui Khoo, Helen Miller, Judith P. Armitage

**Affiliations:** a Department of Biochemistry, University of Oxford, South Parks Road, Oxford, United Kingdom; b Clarendon Laboratory, Department of Physics, University of Oxford, Oxford, United Kingdom; The Ohio State University

**Keywords:** CheY, cytoplasmic crowding, diffusion, FRET, *Rhodobacter sphaeroides*, single molecule microscopy, vesicles

## Abstract

The bacterial cytoplasm is a very crowded environment, and changes in crowding are thought to have an impact on cellular processes including protein folding, molecular diffusion and complex formation. Previous studies on the effects of crowding have generally compared cellular activity after imposition of stress. In response to different light intensities, in unstressed conditions, Rhodobacter sphaeroides changes the number of 50-nm intracytoplasmic membrane (ICM) vesicles, with the number varying from a few to over a thousand per cell. In this work, the effects of crowding induced by ICM vesicles in photoheterotrophic R. sphaeroides were investigated using a fluorescence resonance energy transfer (FRET) sensor and photoactivated localization microscopy (PALM). In low light grown cells where the cytoplasm has large numbers of ICM vesicles, the FRET probe adopts a more condensed conformation, resulting in higher FRET ratio readouts compared to high light cells with fewer ICM vesicles. The apparent diffusion coefficients of different sized proteins, PAmCherry, PAmCherry-CheY_6_, and L1-PAmCherry, measured via PALM showed that diffusion of protein molecules >27 kDa decreased as the number of ICM vesicles increased. In low light R. sphaeroides where the crowding level is high, protein molecules were found to diffuse more slowly than in aerobic and high light cells. This suggests that some physiological activities might show different kinetics in bacterial species whose intracellular membrane organization can change with growth conditions.

## INTRODUCTION

The bacterial cytoplasm is a highly crowded, diverse environment. The general lack of organizing internal membranes means the bacterial cytoplasm contains both large assemblies of protein complexes and nucleic acids, some localized to specific regions of the cell, plus diffusing substrates and enzymes, ribosomes, and RNA. The packing of biomolecules and the complex architecture of the bacterial cytoplasm lead to macromolecular crowding and low water activity, which means that small changes can affect cellular processes such as diffusion, folding, and association of proteins. Previously, macromolecular crowding in live cells has been estimated by studying the motions of fluorescently labeled molecules in the cytoplasm and the relationship between crowding and diffusion studied by inducing cellular changes that promote or reduce the formation of cytoplasmic barriers and therefore might restrict molecular diffusion. Most of these studies involved the use of stressed conditions such as osmotic upshift (1–3), antibiotic treatments (4–5), and nutrient limitation (6) to allow comparisons between the perturbed and the native state of the bacterial population. However, these treatments may lead to substantial changes in the cell’s physiology and complex adaptation mechanisms that also affect cellular diffusion. Therefore, we set out to study macromolecular crowding and its effects on protein diffusion in a physiologically versatile bacterium under different growth conditions known to naturally change the cellular organization.

Rhodobacter sphaeroides is an alphaproteobacterium and a model organism for studying photosynthesis (7) and complex signaling pathways such as chemotaxis (8). Interestingly, aerobically and photoheterotrophically grown R. sphaeroides cells have distinctive cell architectures. When R. sphaeroides is grown aerobically in the dark, it has a cytoplasmic morphology that is similar to E. coli with an uninvaginated inner membrane; however, when the bacterium is grown under photosynthetic conditions, membrane invaginations and 30–70-nm cytoplasmic vesicles (9–10) containing the photosynthetic apparatus fill the cytoplasm. Previous study showed that R. sphaeroides adapts to different levels of light intensity by generating different numbers of intracytoplasmic membrane (ICM) vesicles, with over a thousand per cell in low light grown populations (9). R. sphaeroides also produces polyhydroxybutyrate storage granules under some growth conditions (11) and alters the ribosome content when grown photoheterotrophically (12). These physiological responses to growth conditions could all have effects on the crowded state of the cytoplasm.

In this study, we show that the presence of ICM vesicles in photoheterotrophic R. sphaeroides leads to macromolecular crowding effects that can be detected *in vivo* by a genetically encoded FRET sensor and reflected through the reduction of protein diffusion rates when compared to high light grown cells. We show here that a higher FRET ratio was observed in cells with high ICM levels (low light) compared to cells with low ICM levels (high light), and using single molecule tracking of PAmCherry, we further show that crowding by ICM vesicles alters the diffusion rate of protein molecules >27 kDa in the cytoplasm of photoheterotrophic R. sphaeroides.

## RESULTS

### Quantification of ICM vesicles in high light and low light cells via BChl extraction.

**FIG 1:**
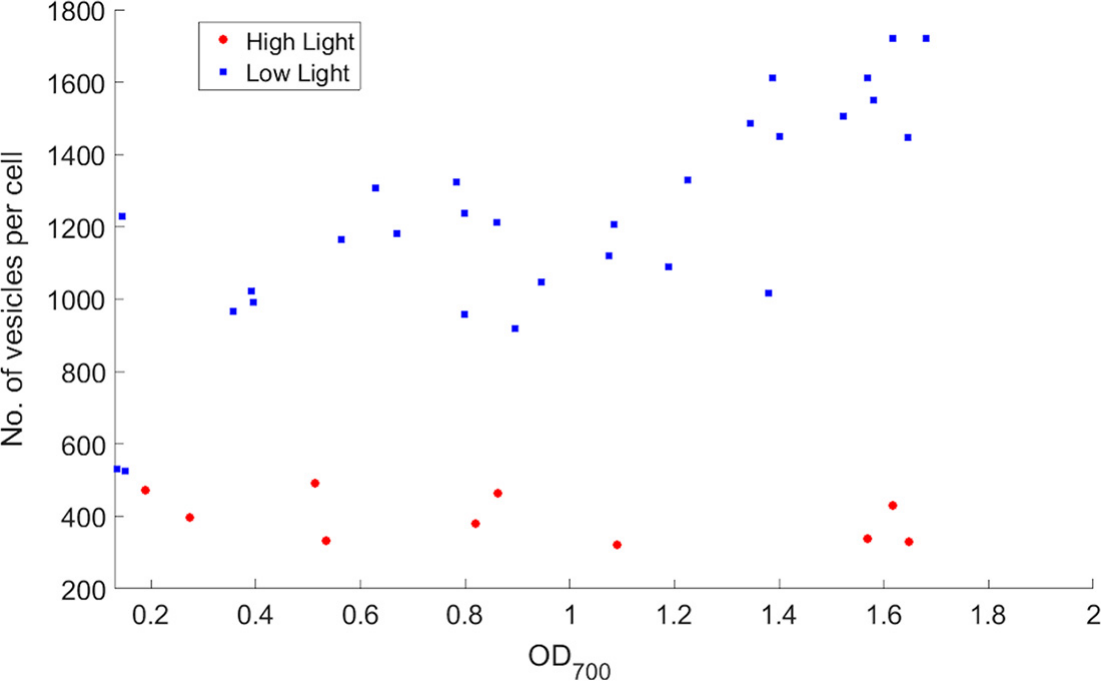
Number of vesicles in photoheterotrophic R. sphaeroides was higher at lower light intensity. The optical density of R. sphaeroides cells was measured at 700 nm, to avoid artifacts caused by absorbance peaks of photosynthetic pigments in photoheterotrophic cells. Photoheterotrophic R. sphaeroides cells were grown under constant illumination without stirring and harvested for BChl extraction. The number of vesicles in cells was estimated by calculating the BChl concentration relative to cell number as described in Materials and Methods. Self-shading probably caused increased vesicle numbers with increasing cell density in cells with high ICM levels, while cells with low ICM levels showed relatively constant vesicle numbers with increasing cell density. The measurements were taken using multiple cultures that were grown to different optical density levels.

R. sphaeroides develops ICM vesicles when grown photoheterotrophically, and the presence of ICM vesicles may induce crowding in the cytoplasm. To measure the number of vesicles in photoheterotrophic R. sphaeroides cells, the bacteriochlorophyll (BChl) concentration relative to cell number was calculated as previously described (see Materials and Methods). The BChl concentration was determined according to the calculations from Adams et al. (9) using the extinction coefficient of 0.076 μM^−1 ^cm^−1^ (13) and converted to vesicle number per cell. Overall, low light cells had approximately three to four times more vesicles than high light cells (Fig. 1). Determining the number of vesicles in R. sphaeroides would help to estimate the proportion of the cytoplasm occupied by ICM vesicles under high light and low light conditions. Low light cells were found to have high ICM levels that could occupy up to 25.1% (0.2312 μm^3^) of the cytoplasm volume, assuming that these low light cells contained 1,700 vesicles per cell on average (Text S1 in the supplemental material).

10.1128/mbio.03672-21.10 TEXT S1 Supplementary text. Download Text S1, DOCX file, 0.02 MB. Copyright © 2022 Khoo et al. 2022 Khoo et al. https://creativecommons.org/licenses/by/4.0/ This content is distributed under the terms of the Creative Commons Attribution 4.0 International license.

### FRET sensor for quantifying macromolecular crowding in photoheterotrophic R. sphaeroides.

**FIG 2:**
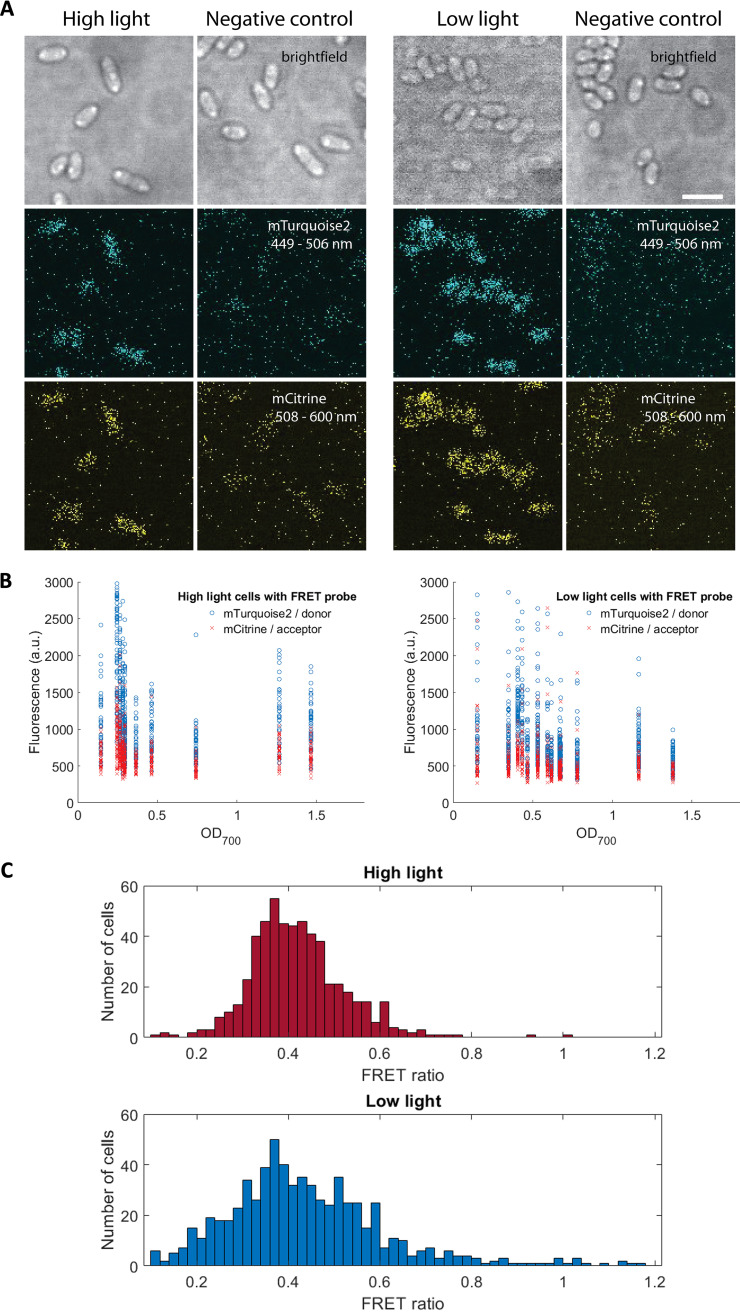
FRET response in photoheterotrophic R. sphaeroides grown in different light intensities. (A) Images of R. sphaeroides cells with high ICM levels (low light) and low ICM levels (high light) expressing the FRET probe from an inducible pIND4 plasmid after excitation by a 405-nm laser. Negative control cells contained a pIND4 plasmid without the FRET probe. Scale bar, 3 μm. (B) The fluorescence distributions of FRET donor mTurquoise2 and acceptor mCitrine emitted by single cells at different OD_700_. (C) The distributions of ratiometric FRET probe ratio for single high light and low light grown R. sphaeroides cells after subtracting the background fluorescence of the negative control. The mean FRET ratio was 0.422 (*n* = 549) for high light grown cells with low ICM levels and 0.477 (*n* = 712) for low light grown cells with high ICM levels. A two-tailed independent *t* test showed that *P* = 0.001722.

Boersma et al. developed a genetically encoded fluorescence resonance energy transfer (FRET) probe to investigate effects of macromolecular crowding in osmotically stressed bacterial and mammalian cells (14–15). The probe consists of a donor and an acceptor protein (mTurquoise2 and mCitrine) pair connected by a flexible linker (15). The FRET signal was measured by dividing the fluorescence signal of acceptor by the signal of donor. When the probe was placed in crowded *in vitro* and *in vivo* environments, it underwent compression and adopted a more condensed conformation, producing an increase in acceptor fluorescence signal and higher FRET signal (14–15). To determine the effects of crowding induced by ICM vesicles, the FRET probe was cloned onto the inducible pIND4 plasmid (16) and expressed in R. sphaeroides (Materials and Methods). Previous study showed that the number of vesicles in R. sphaeroides depends on light intensity (9). Therefore, in this study, R. sphaeroides cells were grown anaerobically under high light and low light condition with 1 mM IPTG induction. Fluorescence microscopy showed that fluorescence signal was evenly distributed in the cytoplasm and the FRET probe was functional in living R. sphaeroides cells (Fig. 2).

Fluorescence images were acquired via confocal microscopy (Materials and Methods). In cells with low ICM levels (*n* = 549), the fluorescence signal was 1139 ± 22 a.u. (mean ± standard error of the mean [SEM]) in the donor channel and 639 ± 9 a.u. in the acceptor channel; whereas in cells with high ICM levels (*n* = 712), the mean ± SEM was 830 ± 20 a.u. in the donor channel and 515 ± 9 a.u. in the acceptor channel. The fluorescence intensity of the probe-containing cells was around 2 to 3 times higher than the background autofluorescence of the negative control in both channels (Fig. S1).

10.1128/mbio.03672-21.1 FIG S1 (A) Fluorescence (a.u.) of low light (LL) and high light (HL) R. sphaeroides in the acceptor (508–600 nm) and donor (449–506 nm) channel. The mean ± standard error of the mean (SEM) was determined using fluorescence intensities taken from Fig. 2B. (B) The fluorescence distributions of background autofluorescence emitted by single photoheterotrophic R. sphaeroides cells at different OD_700_ were lower than the fluorescence emitted by cells containing the FRET probe shown in Fig. 2B. Negative control cells contained a pIND4 plasmid without the FRET probe. Download FIG S1, TIF file, 1.1 MB. Copyright © 2022 Khoo et al. 2022 Khoo et al. https://creativecommons.org/licenses/by/4.0/ This content is distributed under the terms of the Creative Commons Attribution 4.0 International license.

The single cell ratiometric FRET ratio in Fig. 2C was calculated by subtracting the background fluorescence of the negative control and dividing the fluorescence signal of acceptor (508–600-nm) by the signal of donor (449–506-nm) from Fig. 2B. Twelve cells with high ICM levels with fluorescence signals that were lower than the negative control were excluded from this analysis to avoid negative values. Single cells with low ICM levels were found to have a mean FRET ratio of 0.422, while cells with high ICM levels had a higher mean FRET ratio of 0.477, indicating a more crowded environment (Fig. 2C). The histograms of uncorrected single cell ratiometric FRET ratios (without subtracting the autofluorescence) are shown in Fig. S2A, B.

10.1128/mbio.03672-21.2 FIG S2 The distributions of uncorrected ratiometric FRET probe for high light and low light R. sphaeroides cells. (A, B) The mean FRET ratio was 0.5782 (*n* = 549) for cells with low ICM levels (high light) and 0.6441 (*n* = 712) for cells with high ICM levels (low light). The fluorescence intensity of the mCitrine channel (508–600 nm) was divided by the intensity of the mTurquoise2 channel (449–506 nm) to determine the FRET ratio of single cells (not accounting for the autofluorescence in negative control cells). (C, D) The FRET ratios over OD_700_ for both growth conditions were fitted with linear least squares regression using MATLAB. FRET ratios for R. sphaeroides cells with low ICM levels remained constant at approximately 0.6 over increasing OD_700_. FRET ratios of R. sphaeroides with high ICM levels increased with OD_700_ (*n* = 712 cells). Download FIG S2, TIF file, 1.8 MB. Copyright © 2022 Khoo et al. 2022 Khoo et al. https://creativecommons.org/licenses/by/4.0/ This content is distributed under the terms of the Creative Commons Attribution 4.0 International license.

### FRET ratio increases with increasing number of vesicles.

**FIG 3:**
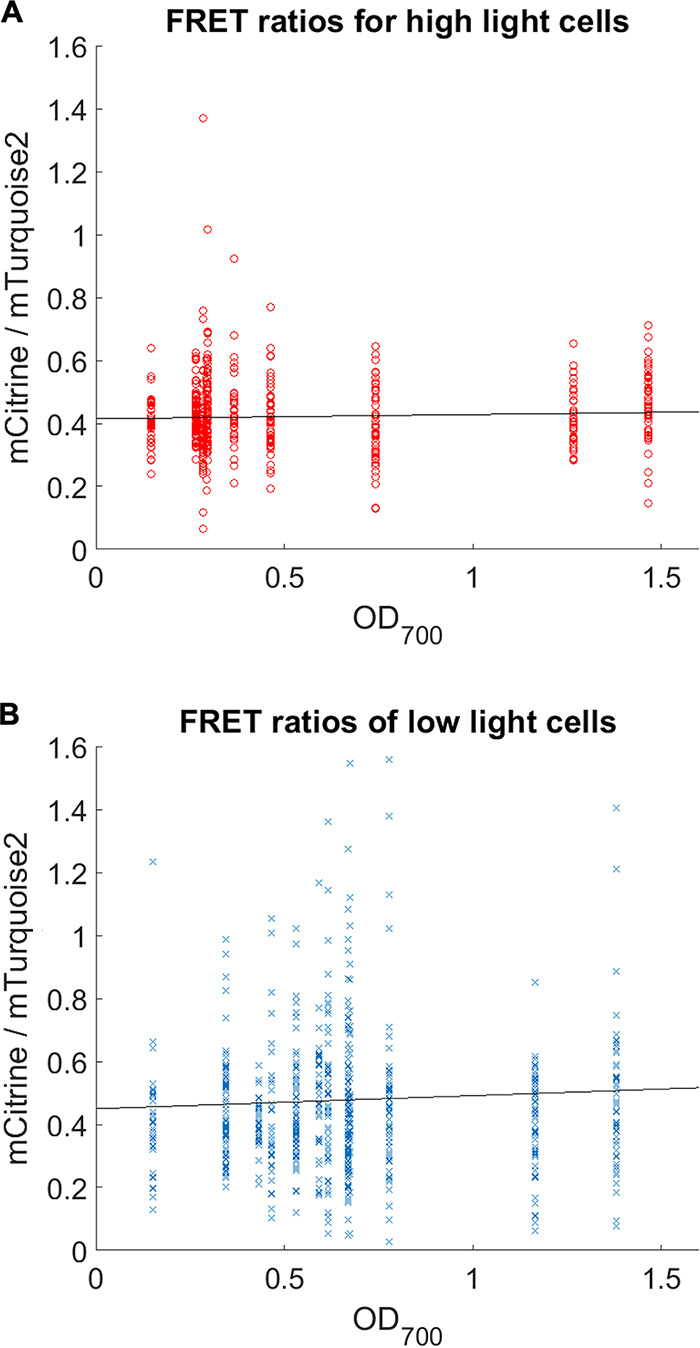
Effect of OD_700_ on FRET ratio for (A) high light grown cells and (B) low light grown cells. The single cell FRET ratio for cells at increasing OD_700_ and therefore ICM numbers was calculated by subtracting the negative control background and dividing the fluorescence intensity of the mCitrine channel (508–600 nm) by the intensity of the mTurquoise2 channel (449–506 nm). The FRET ratios over OD_700_ for both growth conditions were fitted with linear least-squares regression using MATLAB, and it reflected the best fit to the data points. As with ICM numbers, cells with low ICM levels show relatively constant FRET ratios while that of cells with high ICM levels are very variable and increases with ICM number. This suggests that the increased vesicle numbers in low light grown cells leads to increased cytoplasmic crowding.

To understand the dependence of crowding on growth phase (measured through OD_700_), single cell FRET ratios over different OD_700_ units were also calculated and plotted on Fig. 3. Despite cell-to-cell variations, a trend of increasing FRET ratios over increasing OD_700_ could be observed in low light grown cells with high ICM levels. Both high and low light grown cultures with high and low ICM levels had an initial FRET ratio of around 0.4 calculated by extrapolation. As OD_700_ increased, the FRET ratio in cells with high ICM levels increased steadily from around 0.4 to 0.5, whereas the ratio in cells with low ICM levels remained constant at approximately 0.4. This contrast in FRET ratio is more obvious during the mid to late exponential phase of the growth curve (OD_700_ > 0.5) when crowding between cells with high and low ICM levels becomes increasingly distinct, supported by findings obtained from BChl extraction (Fig. 1). A clear trend of increasing FRET ratios over increasing OD_700_ in cells with high ICM levels could also be observed in uncorrected FRET ratios (Fig. S2C, D). These results demonstrate that changes in vesicles lead to changes in FRET signal consistent with changes in crowding.

### Two subpopulations of PAmCherry molecules exist in photoheterotrophic cells.

The effects of crowding induced by ICM vesicles in photoheterotrophic R. sphaeroides on protein diffusion were investigated using photoactivated localization microscopy (PALM). PAmCherry (17), a 27 kDa fluorescent protein, was selected as the fluorescent probe for PALM to avoid overlap with the R. sphaeroides autofluorescence peak. The diffusion of free PAmCherry itself was measured in all cell types. PAmCherry is assumed to diffuse freely and not interact with any cytoplasmic components.

**FIG 4:**
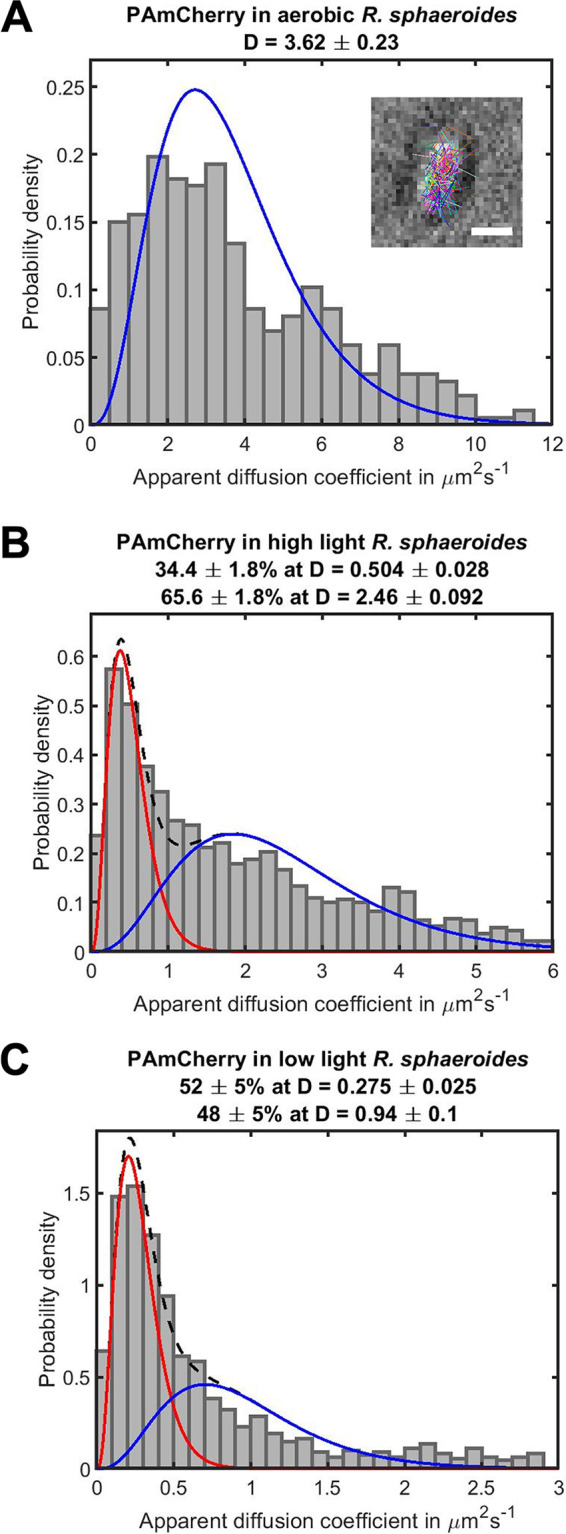
The diffusion profiles of unbound PAmCherry molecules in cells that lack ICM (aerobic) (*n* = 374 molecules), cells with low ICM levels (high light) (*n* = 1751 molecules), and R. sphaeroides cells with high ICM levels (low light) (*n* = 1188 molecules). Note the different *x* axes scales. The diffusion coefficient (D) values were calculated using all tracks with 4 steps (longer trajectories were truncated) and fitted with a least-squares curve; the number of populations was determined by minimizing the reduced χ^2^ statistic (materials and methods). (A) The apparent D values of PAmCherry in cells that lack ICM were combined and fitted to a single gamma distribution for one diffusing species. An example brightfield image of cell overlaid with single-molecule tracks shown in (A), scale bar 1 μm. (B, C) Two diffusing species were observed in high light and low light grown R. sphaeroides cells; therefore, PAmCherry apparent D values for photoheterotrophic cells were fitted with two gamma distributions. Red curve: first diffusing species (the top apparent D value). Blue curve: second diffusing species (the bottom apparent D value). Black dashed curve: sum of distributions of both diffusing species.

The calculated apparent diffusion coefficients (D) of PAmCherry in E. coli (Fig. S4) and aerobic R. sphaeroides that lack ICM (Fig. 4A) were 3.53 ± 0.13 μm^2^ s^−1^ and 3.62 ± 0.23 μm^2^ s^−1^, respectively. The standard errors on the fitted D values were determined by bootstrapping.

10.1128/mbio.03672-21.4 FIG S4 The apparent diffusion coefficients D of unbound PAmCherry molecules in E. coli (*n* = 847 molecules). The D values were calculated using all tracks with 4 steps (longer trajectories were truncated) and fitted with a least-squares gamma distribution. Scale bar, 1 μm. In this work, the diffusion coefficient of PAmCherry in *E.coli* via PALM was found to be 3.53 ± 0.13 μm^2^ s^−1^, which is on the same order of magnitude as the diffusion coefficients of GFP in bacterial cytoplasm measured by other techniques (D = 3–14 μm^2^s^−1^) (1, 2, 27, 28). This indicates that higher-speed particles are not missed by the imaging and tracking procedure. Download FIG S4, TIF file, 0.6 MB. Copyright © 2022 Khoo et al. 2022 Khoo et al. https://creativecommons.org/licenses/by/4.0/ This content is distributed under the terms of the Creative Commons Attribution 4.0 International license.

By analyzing the diffusion profiles of PAmCherry in individual R. sphaeroides cells with high ICM levels, a small percentage of the population was found to have mean D of around 3.3 μm^2^ s^−1^ (similar to the diffusion in cells that lack ICM), while the majority of the cells had a lower mean D of around 1–2 μm^2^ s^−1^ (Fig. S5). Two diffusing species were observed in high light grown cells with low ICM levels, and the PAmCherry D values were fitted poorly to a single gamma distribution for one diffusing species. Therefore, the PAmCherry apparent D data for cells with low ICM levels were fitted with two gamma distributions showing a larger fraction (65.6%) of fast diffusing molecules, D = 2.46 ± 0.09 μm^2^ s^−1^, and smaller fraction (34.4%) of slow diffusing molecules, D = 0.50 ± 0.03 μm^2^ s^−1^ (Fig. 4B).

10.1128/mbio.03672-21.5 FIG S5 Different diffusive behaviors of PAmCherry observed in three representative high light cells. In (A), the diffusion rate was slower with a mean D of 1.13 μm^2^s^−1^. The cell in (B) had a mean D of 3.3 μm^2^s^−1^ that was similar to the measured diffusion in aerobic cells. (C) A cell with a mean D of 2.49 μm^2^s^−1^, possibly containing a mixture of both the fast and slow diffusing species. Scale bar, 1 μm. Download FIG S5, TIF file, 0.9 MB. Copyright © 2022 Khoo et al. 2022 Khoo et al. https://creativecommons.org/licenses/by/4.0/ This content is distributed under the terms of the Creative Commons Attribution 4.0 International license.

Two diffusing species were also observed in the PAmCherry D distribution for cells with high ICM levels. In contrast to the result of cells with low ICM levels, around half of the PAmCherry (52%) was diffusing with a much lower D of 0.28 ± 0.03 μm^2^ s^−1^, while 48% had D = 0.94 ± 0.10 μm^2^ s^−1^ (Fig. 4C). The observation that there are two populations of diffusing molecules is intriguing, and while beyond this study it suggests some regions of the cytoplasm may be less crowded than others, allowing access to different sized molecules.

### Diffusion of PAmCherry-Y_6_ in R. sphaeroides.

CheY_6_ is a 14 kDa, well-studied response regulator protein that is known to diffuse freely in the cytoplasm and interact with various chemosensory components localized to different regions of the R. sphaeroides cell, specifically the cytoplasmic chemosensory protein cluster and the flagellar motor. We therefore investigated whether crowding induced by ICM vesicles has an impact on the diffusion and spatial organization of CheY_6_ molecules in R. sphaeroides. PAmCherry-Y_6_ was expressed from the pIND4 plasmid because previous study suggested that the integration of the fluorophore tagged gene into the genome affects the expression of the native operon (18). To assess the functionality of PAmCherry-Y_6_, the 41 kDa CheY_6_ fusion was constructed and expressed from pIND4 in a nonmotile CheY_6_(D56N) mutant (18) background. A swim plate assay showed that PAmCherry-Y_6_ was able to recover the motility and chemotactic ability of the nonmotile strain, showing it could interact with both the cytoplasmic chemosensory cluster and the flagellar motor (Fig. S8).

10.1128/mbio.03672-21.8 FIG S8 Swim plates showed that CheY_6_ fusions are functional and can recover motility in R. sphaeroides. Previous studies have shown N- and C-terminal CheY_6_ genomic fusions to YFP are nonchemotactic (8); therefore, N-terminal CheY_6_ fusions were introduced in the inducible pIND4 plasmid in a nonmotile CheY_6_(D56N) mutant background. Functional CheY_6_ would allow motility by displacing the D56N mutant protein and if phosphorylated would also allow chemotaxis. The motility and chemotactic were analyzed using swim plate assays. The top diagram shows an example of a swim plate spotted with wild-type WS8N (positive control), CheY_6_(D56N) (nonmotile negative control), pIND-PAGFP-Y_6_, and PAmCherry-Y_6_ in CheY_6_(D56N) background. The colony diameters were measured after 48 h incubation under 100 μM IPTG induction. The bottom diagram shows the average colony diameters of strains expressing CheY_6_ fusions compared to positive and negative control. The strain containing pIND-PAmCherry-Y_6_ recovered chemotaxis with the addition of 10 μM IPTG. The pIND-PAmGFP-Y_6_ strain had comparable swim diameters to the wild type during 100 μM IPTG induction. Two repeats were performed in triplicates. The error bars refer to the standard error. Download FIG S8, TIF file, 0.4 MB. Copyright © 2022 Khoo et al. 2022 Khoo et al. https://creativecommons.org/licenses/by/4.0/ This content is distributed under the terms of the Creative Commons Attribution 4.0 International license.

**FIG 5:**
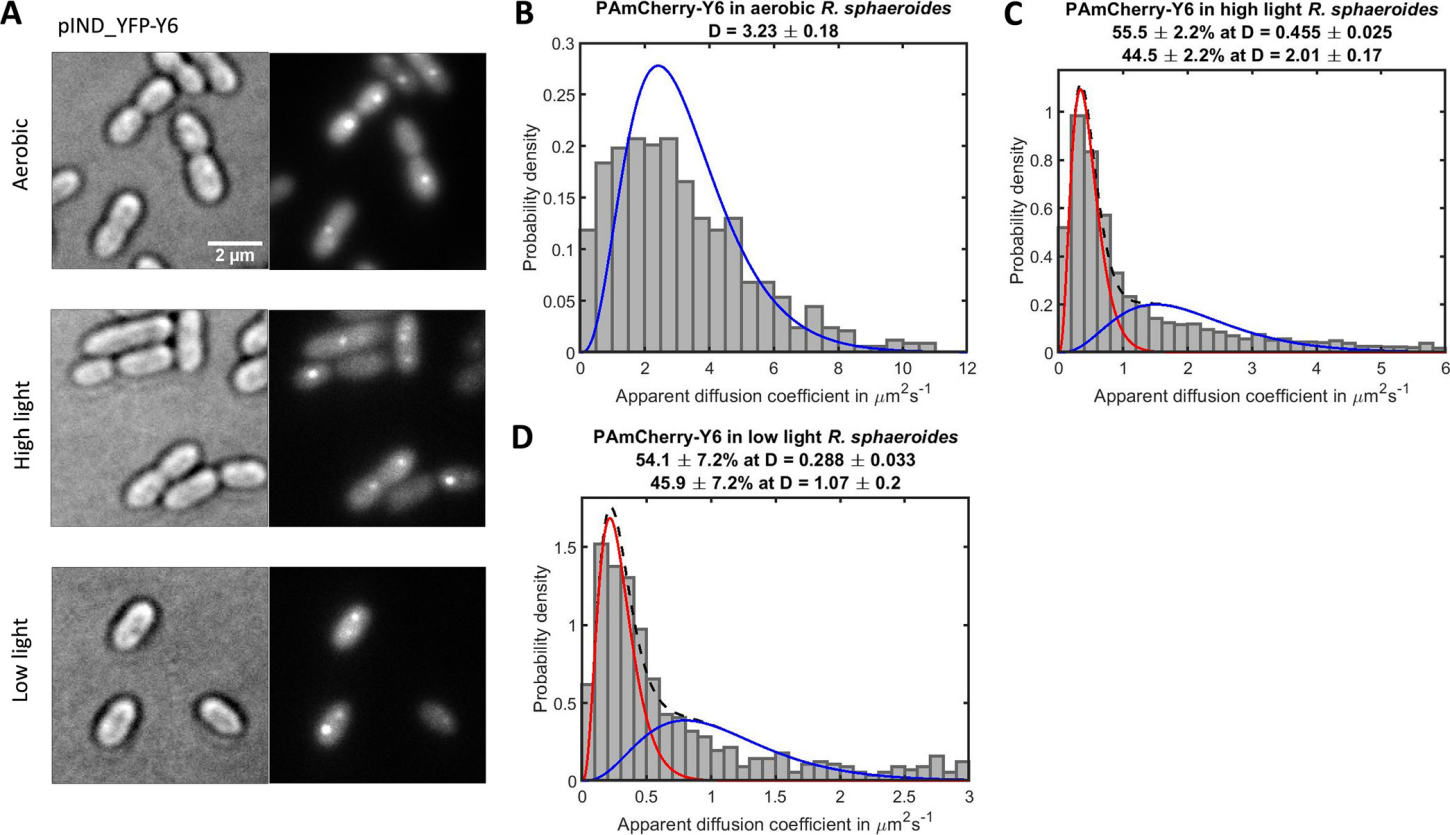
Spatial distribution and diffusion coefficient distributions of YFP-Y_6_ in aerobic and photoheterotrophic R. sphaeroides expressed from the inducible pIND4 plasmid. (A) Spatial distribution of YFP-Y_6_. 81.4% of aerobic cells have a CheY_6_ spot at the cytoplasmic cluster (*n* = 113 cells). For photoheterotrophic cells, 88.9% high light grown cells (*n* = 180 cells) and 79.2% low light grown cells have a fluorescent spot (*n* = 149 cells) in addition to the diffuse fluorescence within the cytoplasm. (B–D) The apparent diffusion coefficients D of PAmCherry-Y_6_ molecules in aerobic R. sphaeroides that lack ICM (*n* = 678 molecules), high light grown R. sphaeroides with low ICM levels (*n* = 2211 molecules), and low light grown R. sphaeroides with high ICM levels (*n* = 624 molecules) on different diffusion scales. The D values were calculated using all tracks with 4 steps (longer trajectories were truncated) and fitted with a least-squares curve; the number of populations was determined by minimizing the reduced χ^2^ statistic (Materials and Methods). Red curve: first diffusing species (the top apparent D value). Blue curve: second diffusing species (the bottom apparent D value). Black dashed curve: sum of distributions of both diffusing species.

This was confirmed by epifluorescence microscopy of YFP-CheY_6_ expressed from pIND4 plasmid, which showed the spatial organization of CheY_6_ in R. sphaeroides under different growth conditions (Fig. 5A). In all growth conditions, the majority of cells had a spot localized at the cytoplasmic chemosensory cluster and diffuse fluorescence throughout the cytoplasm (Fig. 5A); cytoplasmic spots at the chemosensory cluster occurred in 81.4% of aerobic cells that lack ICM (*n* = 113), 88.9% of high light cells with low ICM levels (*n* = 180), and 79.2% of low light cells with high ICM levels (*n* = 149). These data show that YFP-Y_6_ molecules have similar spatial organization regardless of growth conditions.

PALM was used to measure the apparent D of PAmCherry-Y_6_ in E. coli and in R. sphaeroides with different crowding levels. In E. coli and R. sphaeroides cells that lack ICM, apparent D of 3.19 ± 0.083 and 3.23 ± 0.18 μm^2^ s^−1^ were obtained for PAmCherry-Y_6_, respectively (Fig. 5B and Fig. S6). Next, the diffusion of PAmCherry-Y_6_ in photoheterotrophic R. sphaeroides was measured (Fig. 5C and D). In cells with low ICM levels, 44.5% PAmCherry-Y_6_ had D = 2.01 ± 0.16 μm^2^ s^−1^, and 55.5% was diffusing with a smaller D of 0.46 ± 0.03 μm^2^ s^−1^. In cells with high ICM levels, which had similar percentages of fast and slow populations, each population was diffusing more slowly; 45.9% was diffusing at D = 1.07 ± 0.20 μm^2^ s^−1^, and 54.1% of the molecules had a much slower D = 0.29 ± 0.03 μm^2^ s^−1^.

10.1128/mbio.03672-21.6 FIG S6 The apparent diffusion coefficients D of PAmCherry-Y_6_ molecules in E. coli (*n* = 1390 molecules). The D values were calculated using all tracks with 4 steps (longer trajectories were truncated) and fitted with a least-squares gamma distribution. Scale bar, 1 μm. Download FIG S6, TIF file, 0.8 MB. Copyright © 2022 Khoo et al. 2022 Khoo et al. https://creativecommons.org/licenses/by/4.0/ This content is distributed under the terms of the Creative Commons Attribution 4.0 International license.

### Diffusion of L1-PAmCherry in R. sphaeroides.

To obtain information about the diffusion of large complexes *in* R. sphaeroides grown under different conditions, single molecule tracking was performed on the R. sphaeroides ribosomal protein L1 labeled with PAmCherry. We aimed to create functional, fluorescently labeled L1 to use as proxies for ribosomes and study the ribosome dynamics in R. sphaeroides under different crowding levels. Unfortunately, all attempts to create L1-YFP and L1-PAmCherry fusions through homologous recombination were unsuccessful; therefore, the protein was overexpressed in a wild-type background as, even if not completely incorporated into the ribosome, the L1 protein is 24 kDa and therefore larger than CheY_6_. Expression of L1 from pIND4 did however lead to a reduction in growth rate and in cell elongation, suggesting that it was incorporated into ribosomes and interfered with protein synthesis.

The D distribution of the 51 kDa L1-PAmCherry showed two diffusing species in E. coli. Two fractions of molecules diffusing at 0.44 ± 0.05 μm^2^ s^−1^ (41.2%) and 1.22 ± 0.12 μm^2^ s^−1^ (58.8%) were observed (Fig. S7A). Previous studies showed that rifampicin inhibits transcription and affects the structure of the nucleoid, causing an increase in the diffusion rate of ribosomes in E. coli (19) and Caulobacter crescentus (20). Therefore, E. coli expressing L1-PAmCherry was treated with rifampicin to investigate its effect on L1 diffusion. Rifampicin treated cells showed the expected increase in diffusion for both the fast and slow diffusing species (Fig. S7B). For the fast diffusing species, the apparent D increased from 1.22 ± 0.12 (wild type) to 2.16 ± 0.10 μm^2^s^−1^ after the rifampicin treatment, probably reflecting the presence of faster molecules with D > 2 μm^2^s^−1^. The slow species also showed a slight increase in D value (from 0.44 ± 0.05 to 0.65 ± 0.05 μm^2^s^−1^), but the significance is unclear due to the high error values.

10.1128/mbio.03672-21.7 FIG S7 (A and B) The apparent diffusion coefficients D of L1-PAmCherry molecules in E. coli (*n* = 1421 molecules) and rifampicin-treated E. coli (*n* = 744 molecules). The D values were calculated using all tracks with 4 steps (longer trajectories were truncated) and fitted with a least-squares curve. (C) The apparent diffusion coefficients D of L1-PAmCherry molecules in rifampicin-treated aerobic R. sphaeroides (*n* = 286 molecules). Download FIG S7, TIF file, 1.6 MB. Copyright © 2022 Khoo et al. 2022 Khoo et al. https://creativecommons.org/licenses/by/4.0/ This content is distributed under the terms of the Creative Commons Attribution 4.0 International license.

**FIG 6:**
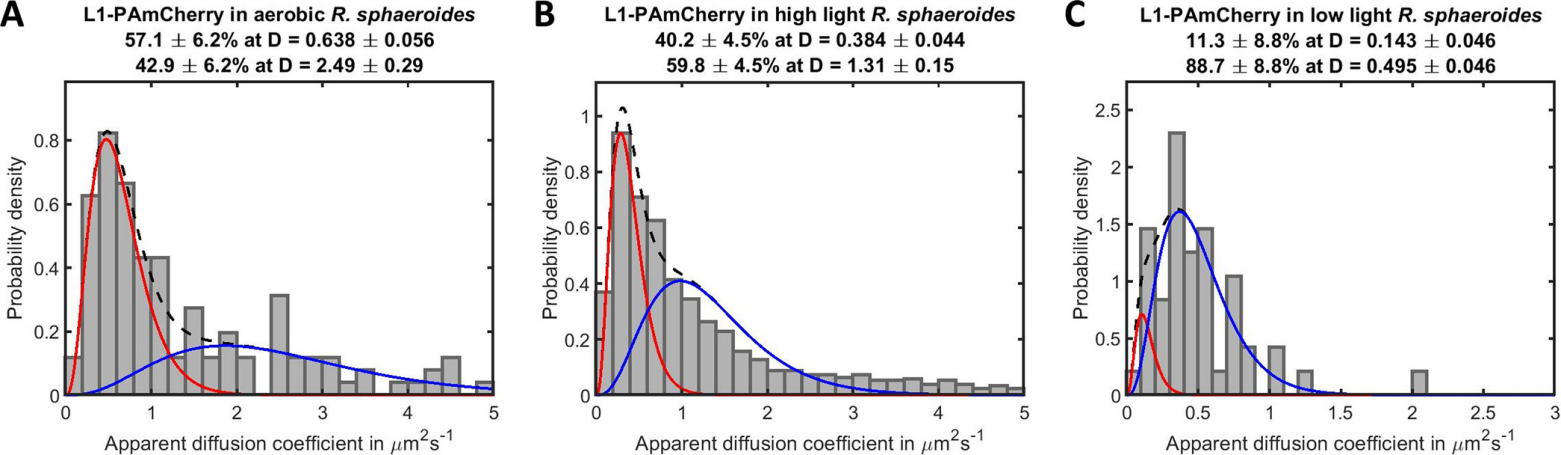
The apparent diffusion coefficients D of L1-PAmCherry molecules in (A) aerobic cells (*n* = 135 molecules), (B) high light grown cells (*n* = 1102 molecules), and (C) low light grown cells (*n* = 50 molecules). Note the different *x* axes scales. Red curve: first diffusing species (the top apparent D value). Blue curve: second diffusing species (the bottom apparent D value). Black dashed curve: sum of distributions of both diffusing species.

In R. sphaeroides cells that lack ICM, 57.1% of L1-PAmCherry molecules had an apparent D of 0.64 ± 0.06 μm^2^ s^−1^, and 42.9% had an apparent D of 2.49 ± 0.29 μm^2^ s^−1^ (Fig. 6A). Upon treatment with rifampicin, there was an increase in the fast fraction from 42.9% to 51.9%, while the slow fraction decreased from 57.1% to 48.1% (Fig. S7C). In contrast to the observed nucleoid-ribosome segregation in E. coli, in the article by Dubarry et al. (21), we showed that the large chromosome of R. sphaeroides is anchored at the poles and therefore occupies much of the cytoplasm, and while the spatial distribution of the ribosomes is unknown, in the related C. crescentus they are found in the chromosomal region. The diffusion of ribosomes in C. crescentus was therefore used as a reference.

Previously, 3D single molecule tracking of L1-YFP in C. crescentus found that the majority of the population (89%) had a D of 0.039 μm^2^s^−1^, while the rest of the population was shown to have a faster D of 3.23 μm^2^s^−1^ (20). After treating the cells with rifampicin, Bayas et al. found that the D of the slow L1 population increased from 0.039 to 0.215 μm^2^s^−1^. The increase in D was similar to the values measured in both E. coli and aerobic R. sphaeroides, and probably reflects the diffusion of incorporated L1 unbound to mRNA.

In photoheterotrophic cells, the histograms of L1-PAmCherry for both photoheterotrophic growth conditions show a shift from fast to slow diffusion compared to aerobic cells, and low light grown cells were found to have two populations of slow diffusing molecules with D < 0.5 μm^2^ s^−1^ (Fig. 6). This again supports the idea that there is increased crowding in low light grown cells as a result of the increase in ICMs and this affects cytoplasmic diffusion.

## DISCUSSION

Using a genetically encoded FRET probe and single molecule tracking, we show that ICM vesicles in photoheterotrophic R. sphaeroides increase crowding in the cytoplasm. ICM vesicles probably act as natural macromolecular crowders increasing the FRET signal of a crowding sensor and decreasing diffusion of proteins > 27 kDa in the cytoplasm of R. sphaeroides.

High light grown cells with low ICM levels were found to have a mean single cell ratiometric FRET ratio of 0.422, while cells with high ICM levels had a mean FRET ratio of 0.477, suggesting that crowding was higher in low light grown cells. BChl extraction estimated 3 to 4 times more vesicles in low light grown cells (around 1,200 vesicles per cell) compared to high light cells (around 400 vesicles per cell). Based on the estimated cell and vesicle volume (Text S1), the ICM vesicles occupy 5.6% of the cell volume in high light grown cells (400 vesicles per cell) and 19.2% of the cell volume in low light grown cells (1,200 vesicles per cell). It is therefore likely that in cells with high ICM levels, a more crowded environment was created by the large number of vesicles, significantly decreasing the cytoplasmic volume.

The effects of ICM vesicles and increased crowding suggested by the FRET measurements on the diffusion of different sized proteins were investigated using single molecule tracking. Our data suggest that ICM vesicles in R. sphaeroides lower the mobility of proteins > 27 kDa with a slow-diffusing population of PAmCherry and PAmCherry-CheY_6_ observed in photoheterotrophically grown cells but absent in aerobic cells. The diffusion rate of the protein was found to decrease by around an order of magnitude between cells lacking ICM and those with high ICM content. The apparent diffusion coefficient of PAmCherry and PAmCherry-CheY_6_ in cells that lack ICM changed from 3.62 ± 0.23 and 3.23 ± 0.18 μm^2^s^−1^, respectively, to around 0.3 μm^2^s^−1^. It is also interesting to note that the addition of a small 14 kDa CheY_6_ caused a significant difference in D between free PAmCherry and PAmCherry-Y_6_. For instance, in high light cells with low ICM levels, the apparent diffusion coefficients of the fast-diffusing population of PAmCherry and PAmCherry-Y_6_ were 2.46 ± 0.09 and 2.01 ± 0.17 μm^2^s^−1^, respectively. It is likely that the attachment of CheY_6_ increases the hydrodynamic radius of the fusion protein compared to PAmCherry, as the molecules have comparable molecular masses. Assuming the fusion forms a dumbbell shaped protein, the hydrodynamic radius could be increased by a factor of up to ∼1.7 by the addition of CheY_6_. The larger ribosomal L1 subunit fused to PAmCherry was also shown to have decreased mobility in photoheterotrophic cells. Our data suggest the L1 subunit was incorporated into the ribosome, so in this case the addition of a relatively small FP tag is unlikely to change the hydrodynamic radius, and therefore diffusion rate, of the fusion compared to the unlabeled molecule. In addition to the changes in the number of ICM vesicles, the levels of other biomolecules such as proteins, RNA, and polyhydroxybutyrate granules may also change depending on the growth conditions of R. sphaeroides. For instance, previous work showed that RNA content in R. sphaeroides was affected by light intensity (12). Therefore, future studies should consider the role of these potential contributors when investigating cytoplasmic crowding in bacteria.

Overall, these results show that ICM vesicles increase crowding in R. sphaeroides, as shown by the reduced mobility of PAmCherry, PAmCherry-Y_6_, and L1-PAmCherry in the cytoplasm in photoheterotrophic grown cells. Previous studies on the effects of changes in cytoplasmic crowding have used stress such as osmotic change to concentrate the cytoplasm (1–6); here, it is a natural result of growth environment with normally growing populations of cells. While the development of large numbers of ICM vesicles may not occur in many bacterial species, several develop inclusions, and in many the volume occupied by nucleic material can change dramatically through the cell cycle. Further studies are required to understand how cellular activities, such as nucleoid division and separation and adaptations might affect protein diffusion and any effects on cellular activity. In addition, excluded volume effects should be also considered in *in vivo* studies investigating protein dynamics and molecular diffusion.

Combined, our data show that ICM vesicles in R. sphaeroides act to increase macromolecular crowding, resulting in increased FRET signal and reduced protein diffusion coefficients from aerobic cells that lack ICM, through photoheterotrophic high light with low ICM levels, to low light cells with high ICM levels. Therefore, R. sphaeroides is an excellent system to study macromolecular crowding without inducing cell stress. Understanding how crowding levels affect protein mobility is important for diffusion-dependent signaling pathways such as chemotaxis. We found a significant reduction in PAmCherry-CheY_6_ diffusion coefficient in crowded photoheterotrophic R. sphaeroides cells, suggesting that the response and adaptation time of chemotaxis may vary between growth states, although the size of the fluorescent tag may have increased the crowding effect observed.

## MATERIALS AND METHODS

### Growth conditions.

R. sphaeroides WS8N cells were grown in succinate medium/SUX medium (22) at 30°C, either aerobically with shaking (225 rpm), or photoheterotrophically in sealed Eppendorfs or medical flats under constant illumination without stirring. A tungsten lamp (Crompton Lamps, 120 W/2600 K warm white, PAR38) and an LED lamp (Luceco Guardian Slimline, 22 W – 300 W/5000 K day light) were used to provide the light source in the light cabinet. The light intensity was measured by a light meter (Skype, SKP 200), and the temperature in the light cabinet was kept at around 30°C. Photoheterotrophic cells were grown (Fig. S9) at either low light (15 μmol photons m^−2^s^−1^) or high light (800 μmol photons m^−2^s^−1^).

10.1128/mbio.03672-21.9 FIG S9 (A and B) Growth curves of Rhodobacter sphaeroides cells with low ICM levels (high light) and cells with high ICM levels (low light). (C) Number of photoheterotrophic R. sphaeroides cells per OD_700_ unit. CFUs per mL at different OD_700_measurements of high light and low light cells were fitted with linear best-fit lines using Microsoft Excel. Low light cells gave a conversion factor of 1.0 × 10^9^ cells/OD_700_ unit, R^2^ = 0.883, while high light cells gave a conversion factor of 8.0 × 10^8^ cells/OD_700_ unit, R^2^ = 0.8442. Download FIG S9, TIF file, 0.7 MB. Copyright © 2022 Khoo et al. 2022 Khoo et al. https://creativecommons.org/licenses/by/4.0/ This content is distributed under the terms of the Creative Commons Attribution 4.0 International license.

E. coli S-17 cells were grown at 37°C in LB medium with 225 rpm shaking.

### FRET sensor expression in R. sphaeroides.

The crTC2 sensor developed by Liu et al. was cloned onto an inducible pIND4 plasmid (16). R. sphaeroides cultures expressing the FRET sensor on pIND4 were grown photoheterotrophically under high and low light in the presence of 1 mM IPTG. A negative control was included with each FRET experiment, using a strain containing an empty pIND4 vector. When the cells reached the exponential phase, chloramphenicol was added at 30 μg/mL to inhibit protein synthesis and the culture was incubated at 30°C with rigorous shaking for 30 min to allow proper folding and maturation of the remaining immature fluorescent protein.

The cells were washed with SUX medium, placed on 1% agarose pads, and imaged using a laser scanning confocal microscope (Zeiss 780). A 405-nm laser was used to excite the donor protein. The emission of the donor and acceptor proteins were split into two channels: 449-506 nm and 508–600 nm. The fluorescence intensity for single cells in both channels were analyzed using ImageJ.

Single cell FRET ratios over different OD_700_ units were calculated by subtracting the background fluorescence of negative control and dividing the fluorescence signal of acceptor (508–600 nm) by the signal of donor (449–506 nm). The data were fitted with linear least-squares regression using MATLAB.

### Bacteriochlorophyll (BChl) extraction using acetone/methanol.

One mL of R. sphaeroides culture grown to the appropriate optical density (OD) was harvested by 13,300 × *g* for 2 min. OD was measured at 700 nm to prevent interference from pigments. The pellet was resuspended in 1 mL of acetone/methanol (7:2 by volume) and centrifuged at 13,300 × *g* for 3 min. The supernatant containing the extracted pigments was collected, and absorbance at 770 nm was measured. The number of photoheterotrophic R. sphaeroides cells in SUX culture was determined by counting the CFU from cell plating experiments. One OD_700_ unit contained approximately 8.0 × 10^8^ high light cells and 1.0 × 10^9^ low light cells, respectively. Quantification of ICM vesicles was calculated using the formula from Adams and Hunter (9).

### PALM.

R. sphaeroides cultures containing the appropriate PAmCherry fusions on pIND4 were grown photoheterotrophically under high and low light in the presence of 1 mM IPTG. Cells were placed on 1% agarose pads and imaged on a custom-built PALM/TIRF microscope at Micron Advanced Imaging Consortium, University of Oxford. The microscope was equipped with an Andor iXon 897 ultra electron multiplying CCD (EMCCD) camera and a Toptica Multi Laser Engine with 405 nm, 488 nm, 561 nm, and 640 nm lasers.

Bright field images of the targeted cells from a 64 × 64 pixels field of view (FOV) were recorded before imaging. The 564-nm laser was used to minimize the cellular autofluorescence. After the prebleach step, the cells were imaged with a 405-nm (336 μW) laser for photoactivation and a 70% (9.5 mW) 564-nm laser for excitation of PAmCherry. The intensity of the 405-nm laser was increased gradually as the PAmCherry molecules in the cells started to photobleach over time. Around 6 movies (800 frames each) were recorded for each FOV. Exposure time was 7 ms, and a cycle time was 7.75 ms. PAmCherry filter set ET600/50 (575–625 nm) was used.

### Single particle tracking analysis using Stormtracker.

Single molecule analysis for the PALM experiment was performed using the automated tracking analysis software Stormtracker (23) (Fig. S3) (with modifications by Matthew Stracy and Helen Miller). Fluorescent spots were localized through a self-defined threshold of 20–40 depending on the signal-to-noise ratio. A tracking window of 8 pixels (pixel size 96 nm) was selected to link 5 localizations together to produce 4-step trajectories. The apparent diffusion coefficient D* = MSD/(4 Δt) was calculated from the mean squared displacement (MSD) formula where Δt is the single-frame image cycle time (7.75 ms).

10.1128/mbio.03672-21.3 FIG S3 Single molecule tracking of free PAmCherry molecules in an E. coli cell using Stormtracker (Text S1). (A) A brightfield image was taken before data acquisition for determining the cell outline. (B) By using a localization intensity threshold of 20, localized spots (blue) were generated in the FOV. The localizations were linked through a tracking window of 8 pixels to form single tracks. (C) An example of a single PAmCherry molecule track (red) in the cell. (D) All tracks with 4 steps or more are shown, color coded by trajectory number. PAmCherry molecules were able to diffuse freely in the cytoplasm. Scale bar, 1 μm. Download FIG S3, TIF file, 1.1 MB. Copyright © 2022 Khoo et al. 2022 Khoo et al. https://creativecommons.org/licenses/by/4.0/ This content is distributed under the terms of the Creative Commons Attribution 4.0 International license.

The diffusion coefficient probability distribution was modeled with a gamma distribution (24–26):

$$\text{F}\left(\text{x},\text{D},\text{N}\right)=\frac{{\left(\frac{N}{\text{D}}\right)}^{\text{N}}{\text{x}}^{\text{N}-1}{\text{e}}^{-\frac{\text{Nx}}{D}}}{\left(\text{N}-1\right)!},$$

where N is the number of independent steps in a track (here, 4) and D is the true diffusion coefficient. The error on the diffusion coefficient is calculated via bootstrapping (27–28); where the full data set comprises n measurements, 0.8n measurements are randomly chosen from the data set and fitted with the gamma distribution. The error on each free parameter in the fit to all n data points is calculated from the standard deviation of the results of 10 bootstrap operations. Multiple gamma distributions can be used to fit data containing multiple diffusing species: the reduced χ^2^ statistic, which includes a penalty for the number free parameters, was used to determine the appropriate number of distributions to fit.

### Swim plates.

R. sphaeroides strains carrying the appropriate plasmids were grown photoheterotrophically in the light cabinet to stationary phase. There were 0.25% agar swim plates made in M22 minimal media with 100 μM attractants (propionate in MiliQ water, 0.22 μm filtered), and IPTG at 0, 10, 100, 1000, or 10000 μM. The plates were inoculated with 5 μL of stationary phase culture as a single drop and incubated at 30°C. Each set was carried out in triplicate, and the swarm diameters were measured after 48 h.
