# Supplementary material for: Measurement of Macromolecular Crowding in Rhodobacter sphaeroides under Different Growth Conditions
Source: mBio. 2022 Jan 25;13(1):e03672-21. doi: 10.1128/mbio.03672-21 (PMC8787474; doi:10.1128/mbio.03672-21)
Supplement: TEXT S1 [file mbio.03672-21-s0001.docx]

**S10. Supplementary Text**

**Estimating the proportion of cytoplasm occupied by vesicles under photoheterotrophic conditions**

To estimate the proportion of the cytoplasm occupied by the vesicles under high light and low light conditions, the cell volume and vesicle volume under low light and high light conditions were determined. The average cell volume (V) for both high light and low light cells were calculated by assuming the cells have the shape of a cylinder capped by two half-spheres [29]: V = πW^2^ (L - W/3)/4, the length (L) and width (W) for low light cells were 1.631 ± 0.038 μm and 0.901 ± 0.016 μm (n = 12 cells), and for high light cells 1.738 ± 0.083 μm and 0.890 ± 0.017 μm (n = 13 cells). The calculated cell volumes were 0.848 μm^3^ for low light cells and 0.897 μm^3^ for high light cells, slightly larger than the previously estimated volume of 0.7 μm^3^ for *R. sphaeroides* cells [10]. Another study estimated the size and volume of single chromatophores using TEM and AFM. Low light and high light *R. sphaeroides* vesicles were found to have a volume of 136 ± 23 × 10^3^ nm^3^ and 126 ± 28 × 10^3^ nm^3^, respectively [9]. Using the estimated vesicle volume from Adams & Hunter, a typical high light cell containing 400 vesicles would have around 0.0504 μm^3^ of the cytoplasmic volume being taken up by the vesicles alone. This corresponded to around 5.6% of the total cytoplasmic volume, assuming that this high light cell contained 400 vesicles and had a cell volume of 0.897 μm^3^. For a low light cell with a volume of 0.848 μm^3^ and contained 1200 vesicles, around 19.2% (0.1632 μm^3^) of the total cell volume would be occupied by the vesicles. As low light cells reached higher optical density, up to 25.1% (0.2312 μm^3^) of the cytoplasm could be occupied by the vesicles, assuming that these low light cells contained 1700 vesicles on average.

**Prebleach to reduce autofluorescence in photoheterotrophic *R. sphaeroides***

PALM utilises a prebleach step to reduce cellular autofluorescence. However, intense laser irradiation, especially at high energy wavelengths (<~460 nm), is damaging to bacterial cells and may lead to cell death and growth arrest. The effect of phototoxicity was investigated by monitoring cell growth after prolonged exposure to the 561 nm laser.

WS8N cells were spotted on SUX medium agarose pads with gridded coverslips and a bright field image was taken. The cells were then exposed to 9.5 mW 561 nm laser illumination for 0, 10, 20, 30 seconds and then allowed to recover photoheterotropically for 12 hours. After recovery, the same cells were re-imaged to determine if cell division was occurring. *R. sphaeroides* cells continue to divide after exposure to <30 s of 561 nm irradiation, but completed fewer divisions than non-bleached cells. Irradiation times >40 s resulted in cells remaining in an 'intact' state without obvious changes in cell morphology or cell lysis, but did not divide in 12h.

**Stormtracker software**

Single PAmCherry spots were localized through a user-defined threshold that was affected by the signal-to-noise ratio. False positive localizations occur when the threshold is too low, conversely, if the threshold is too high, it may lead to false negatives or biasing of slow molecules. A relatively low threshold was selected for tracking PAmCherry molecules due to the low photon count of the fluorophore, however, localized spots were linked into trajectories of at least 4 frames using a tracking window of 8 pixels, which excludes single-frame noise events from the analysis. The tracking window sets the upper limit on how far a molecule can diffuse in one frame. Since fluorescent protein has a fast diffusion coefficient [1-2, 30-31], a relatively large tracking window of 0.768 um was selected to avoid splitting of a single molecular trajectory. An example of the found localisations and reconstructed trajectories using a localisation intensity threshold of 20 is shown in Figure S3.

The apparent diffusion coefficient output by the software provides useful insights regarding the relative diffusive behaviours of molecules in different environments, but do not account for the localization precision, which would indicate how much of the diffusion is attributed to noise in the collected image. Localization precision can be obtained measuring the apparent diffusion coefficient of stationary PAmCherry molecules (bound to an immobile structure or component) in live cells under the same image acquisition settings, but is likely to be a relatively small correction for the proteins investigated in this work.

**References for Supplementary Text**

1. Volkmer B and Heinemann H. 2011. Condition-dependent cell volume and concentration of *Escherichia coli* to facilitate data conversion for systems biology modeling. *Plos One*, 6(7):23126.
2. Mullineaux CW, Nenninger A, Ray N, and Robinson C. 2006. Diffusion of green fluorescent protein in three cell environments in *Escherichia coli*. *J. Bacteriol*., 188(10):3442–3448.
3. Elowitz MB, Surette MG, Wolf PE, Stock JB, and Leibler S. 1999. Protein mobility in the cytoplasm of *Escherichia coli*. *J. Bacteriol*., 181(1):197- 203.
