# Supplementary figures and images for: Measurement of Macromolecular Crowding in Rhodobacter sphaeroides under Different Growth Conditions
Source: mBio. 2022 Jan 25;13(1):e03672-21. doi: 10.1128/mbio.03672-21 (PMC8787474; doi:10.1128/mbio.03672-21)

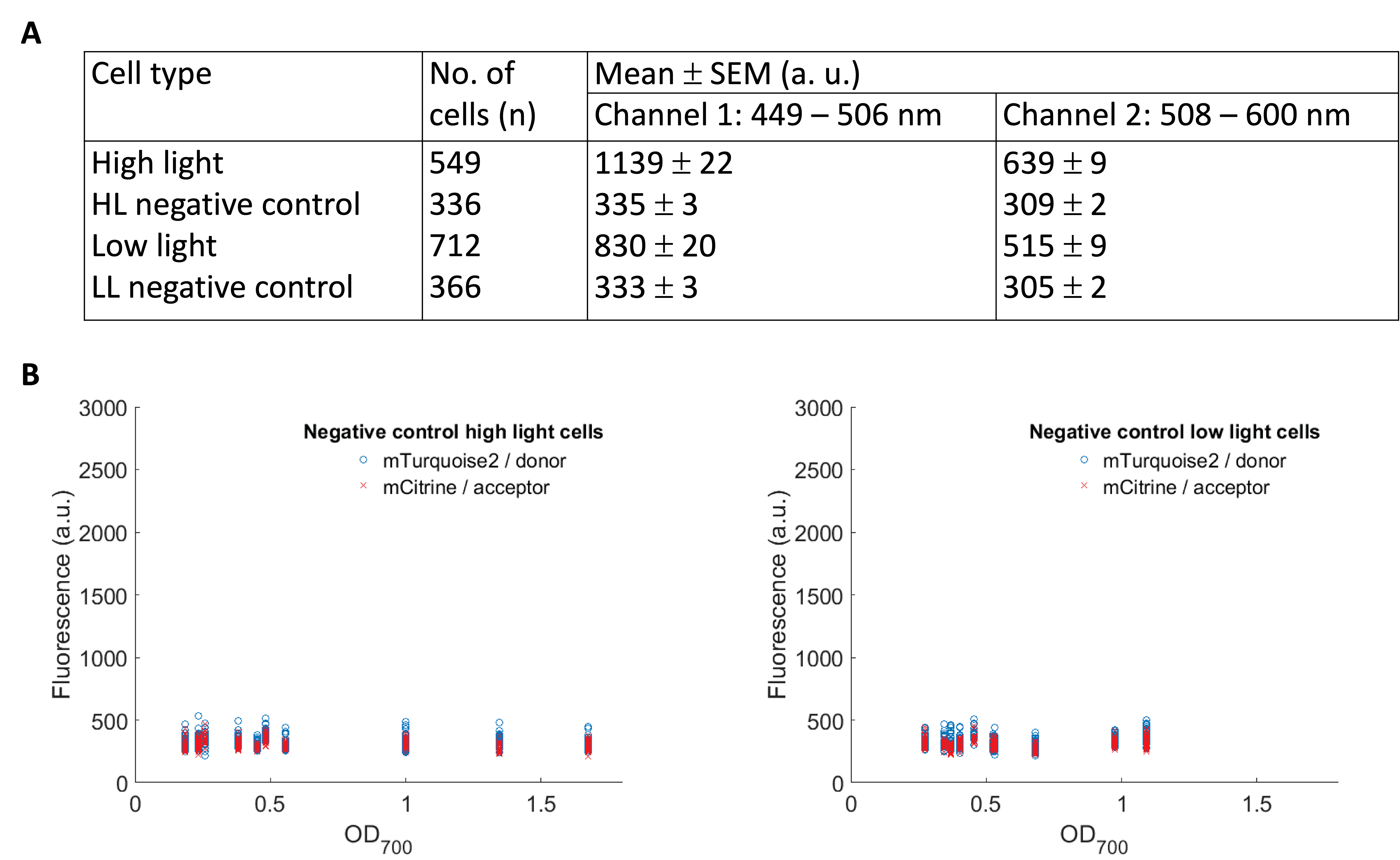

Supplement: FIG S1 [file mbio.03672-21-sf001.tif]

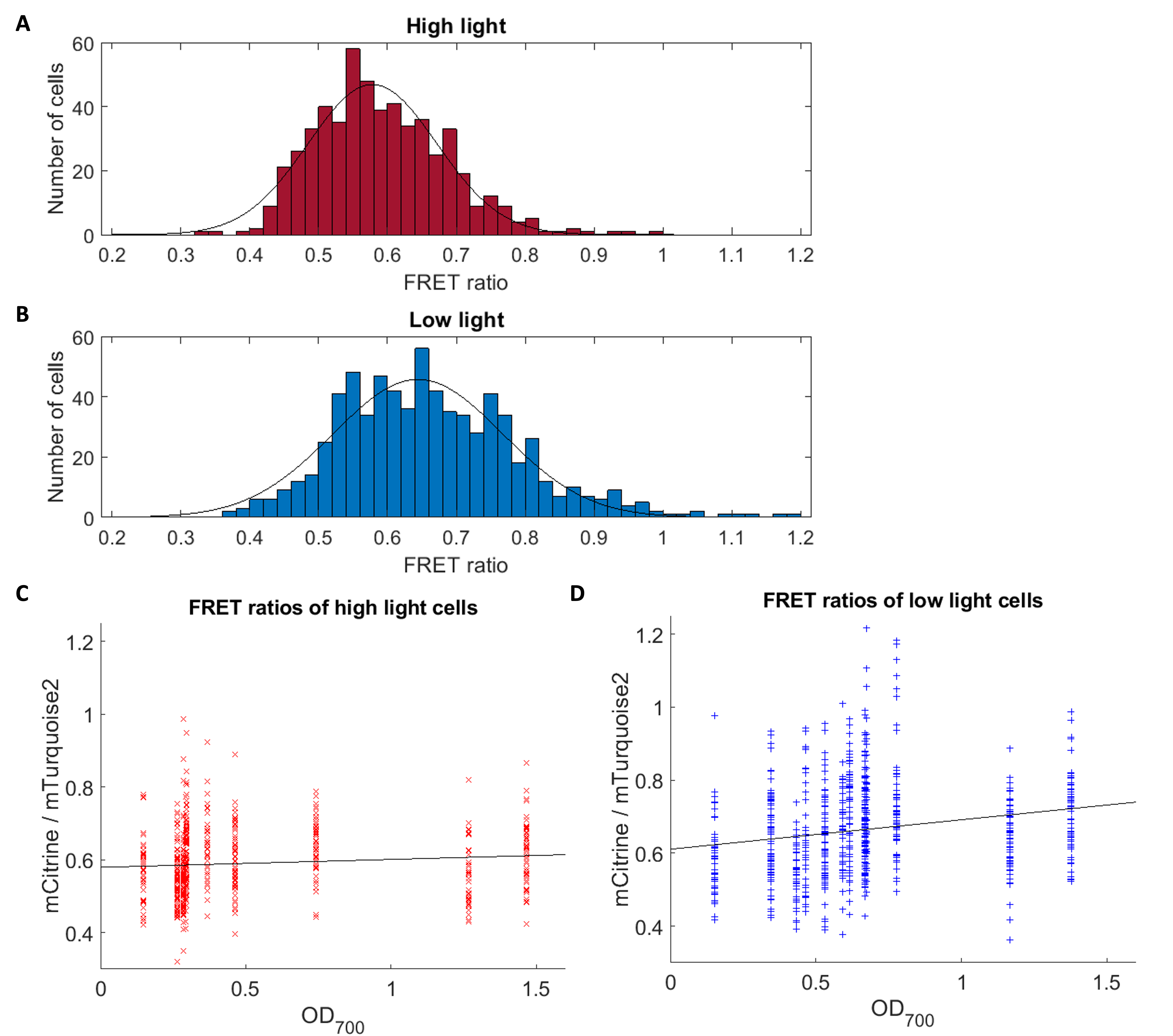

Supplement: FIG S2 [file mbio.03672-21-sf002.tif]

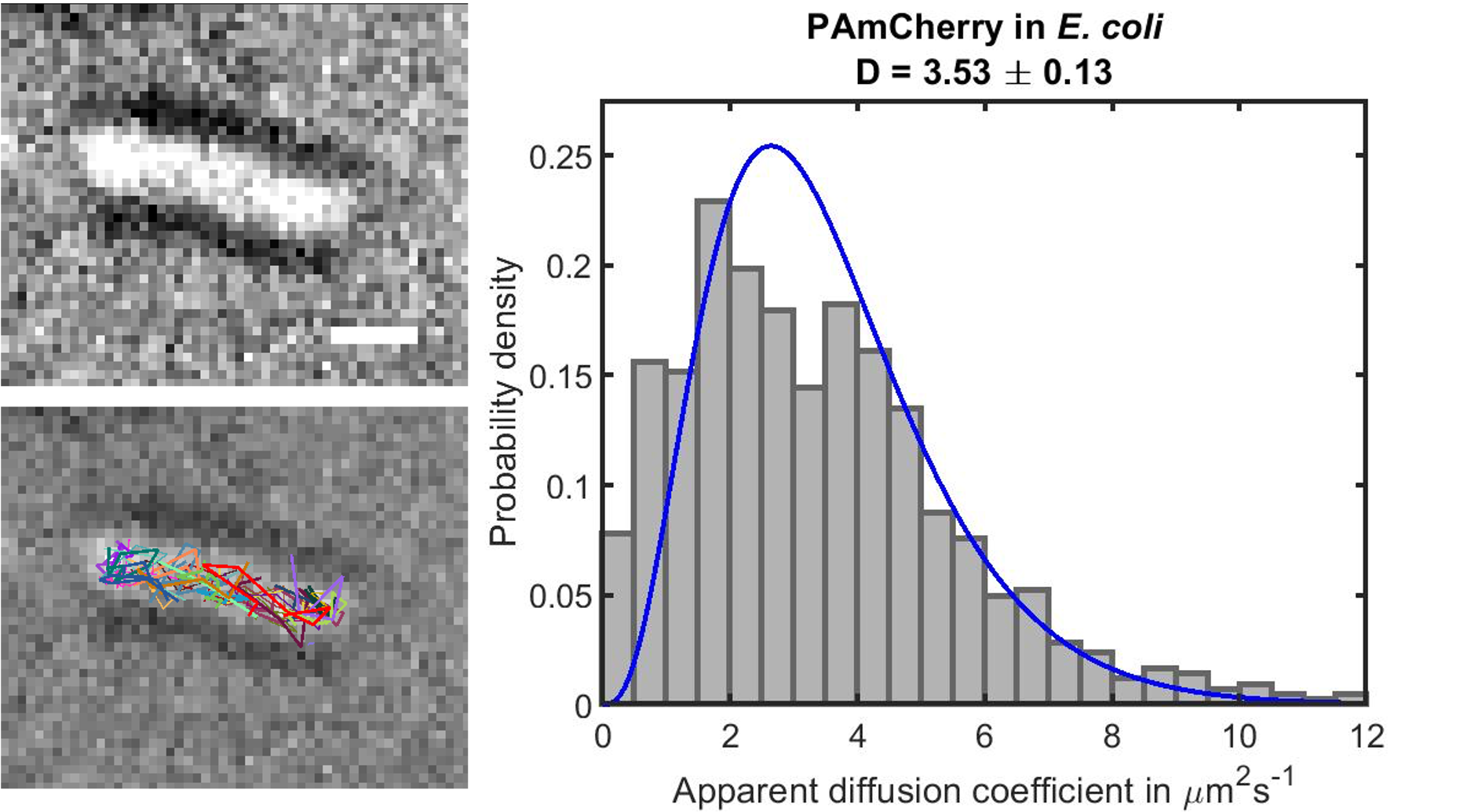

Supplement: FIG S4 [file mbio.03672-21-sf004.tif]

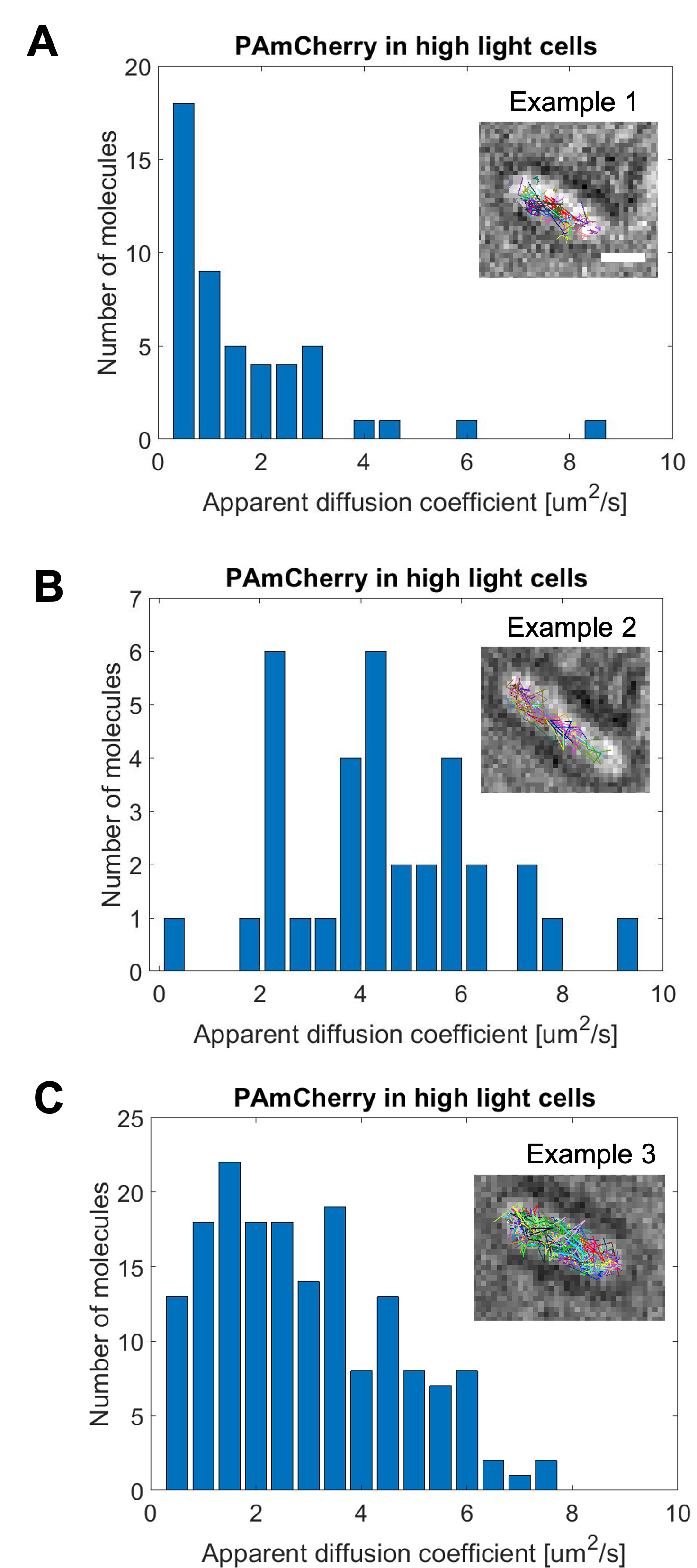

Supplement: FIG S5 [file mbio.03672-21-sf005.tif]

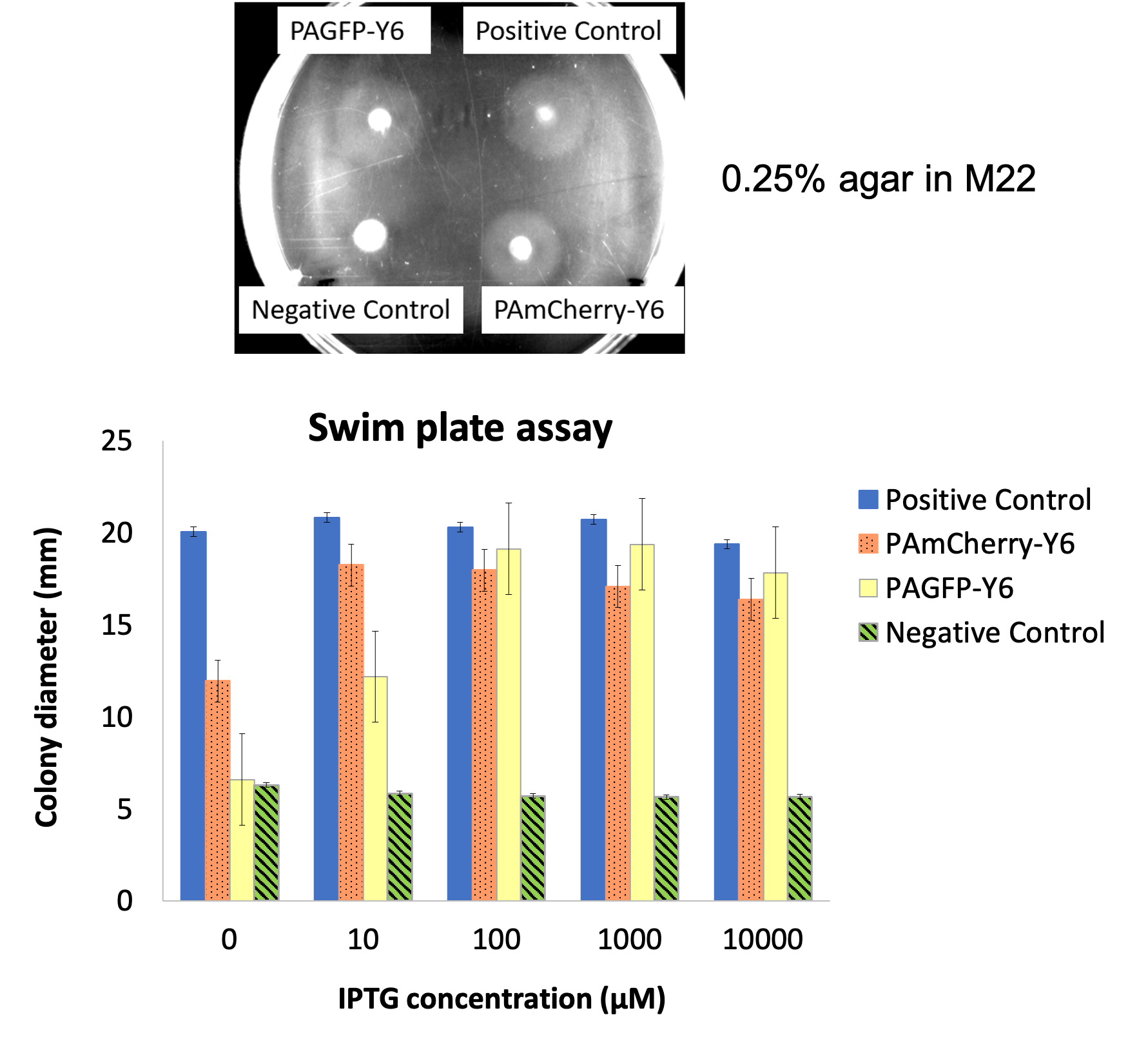

Supplement: FIG S8 [file mbio.03672-21-sf008.tif]

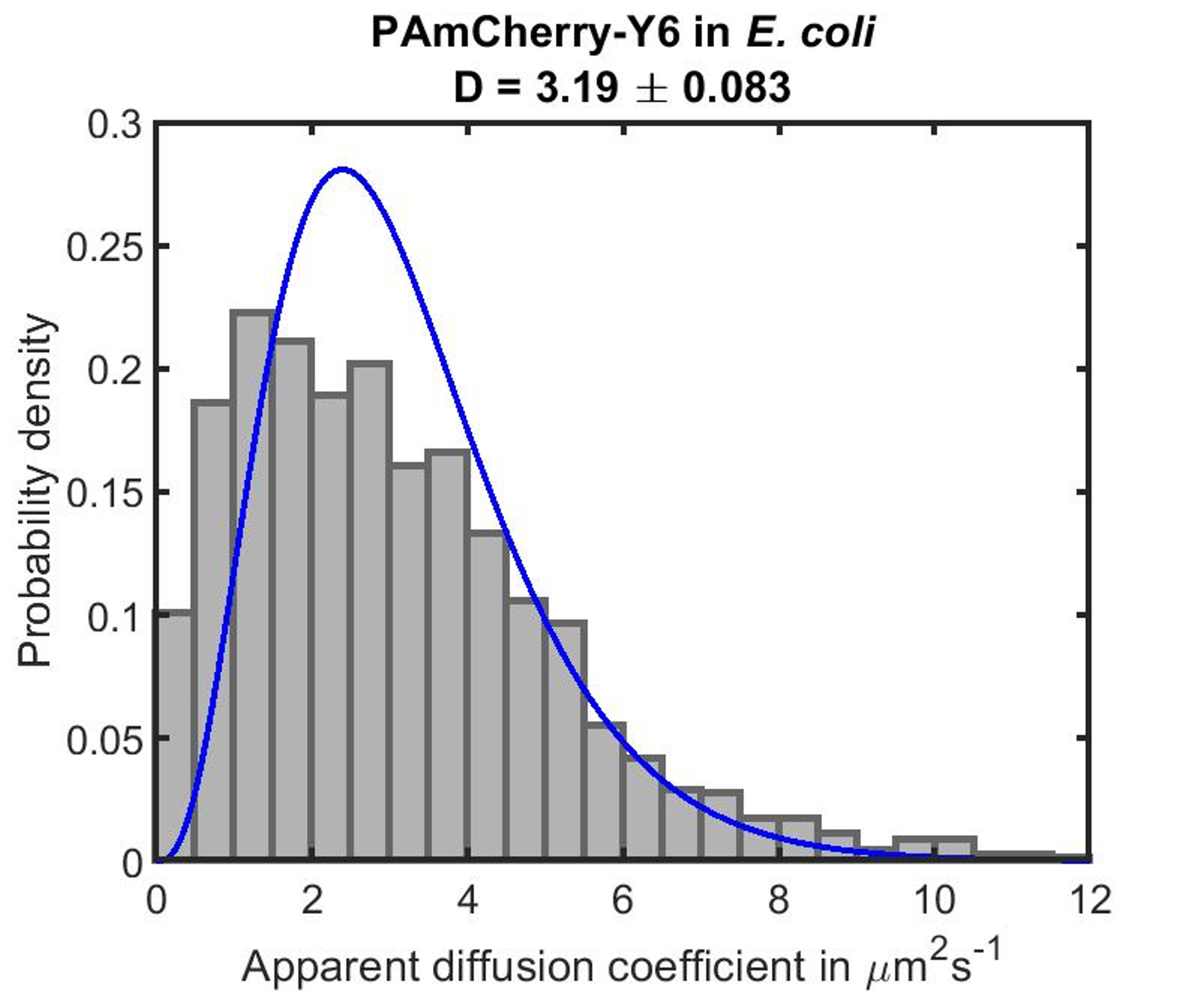

Supplement: FIG S6 [file mbio.03672-21-sf006.tif]

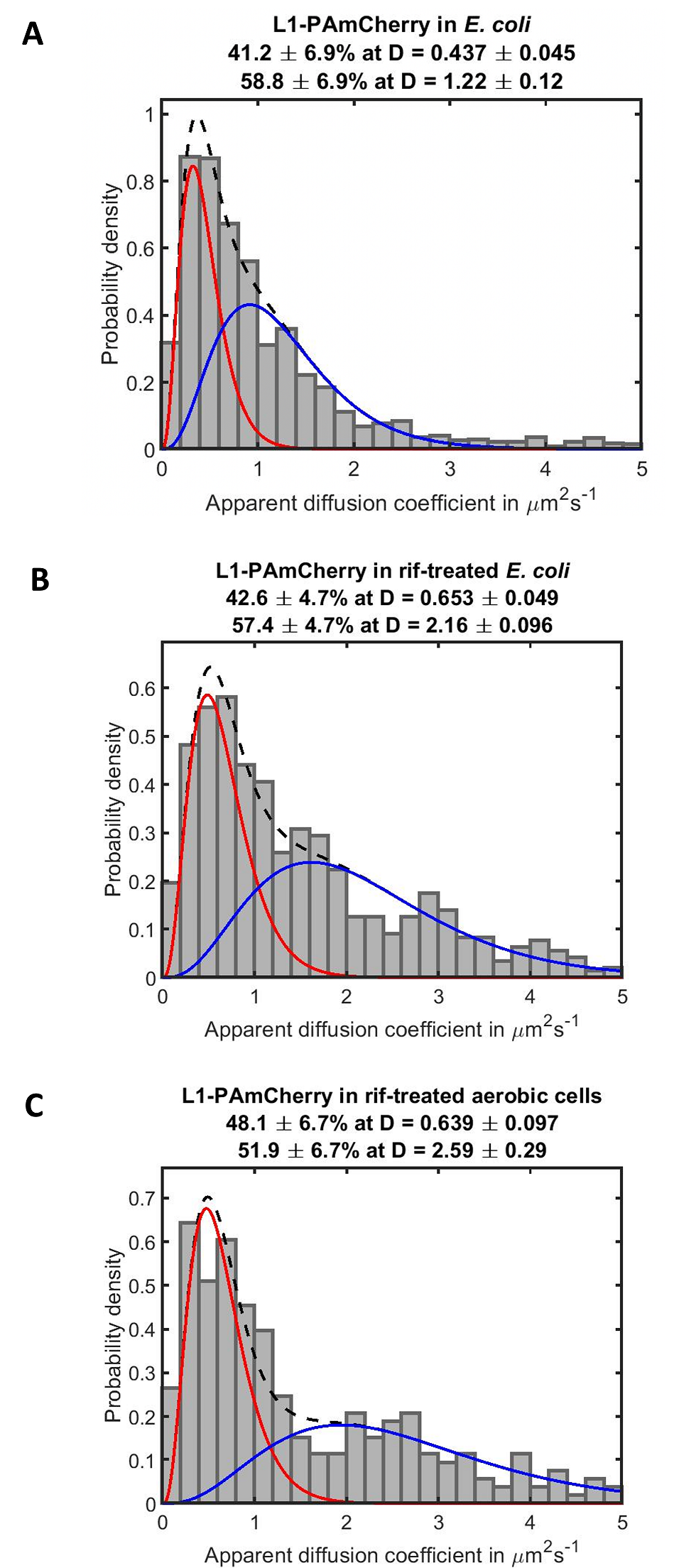

Supplement: FIG S7 [file mbio.03672-21-sf007.tif]

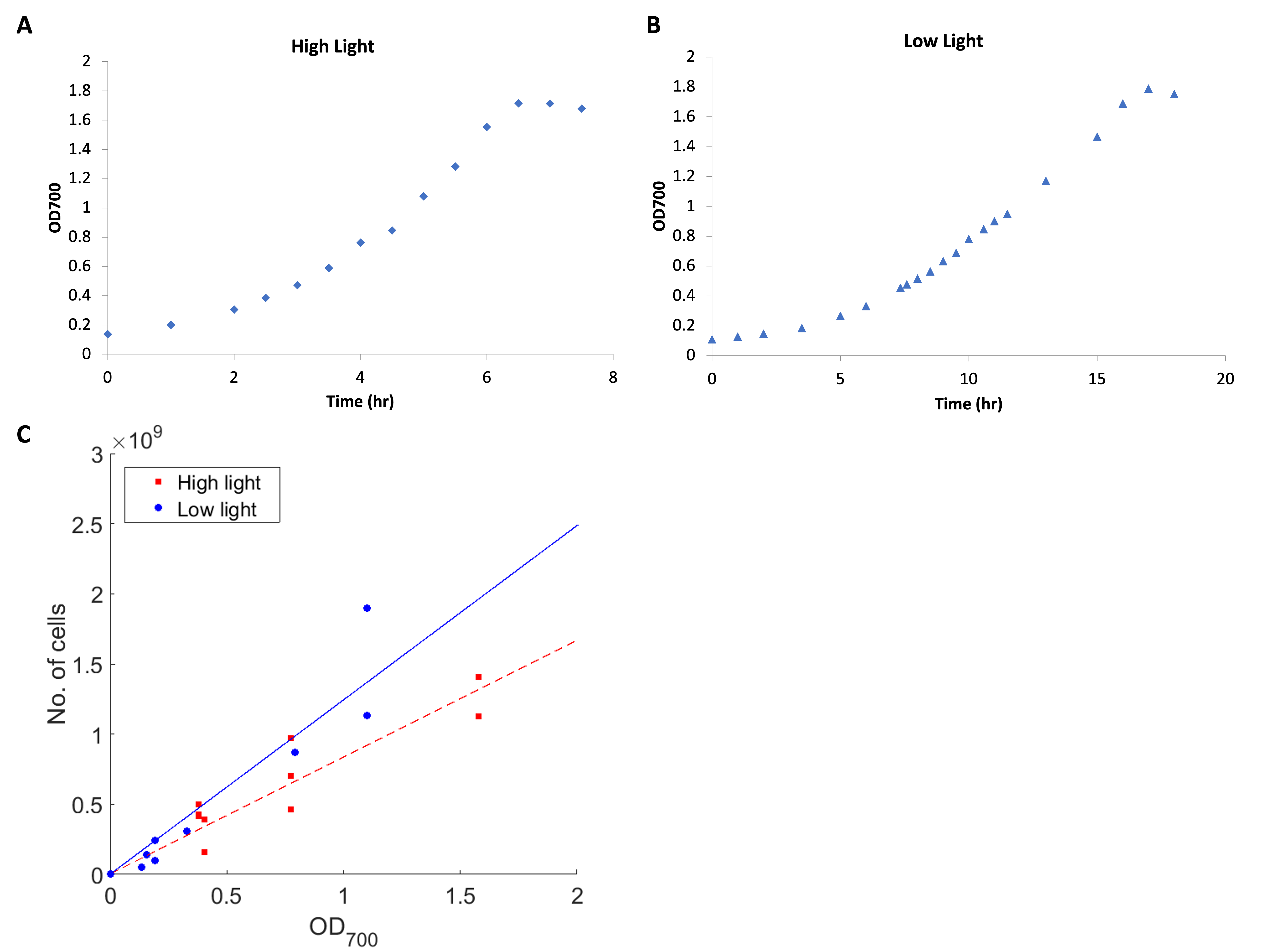

Supplement: FIG S9 [file mbio.03672-21-sf009.tif]

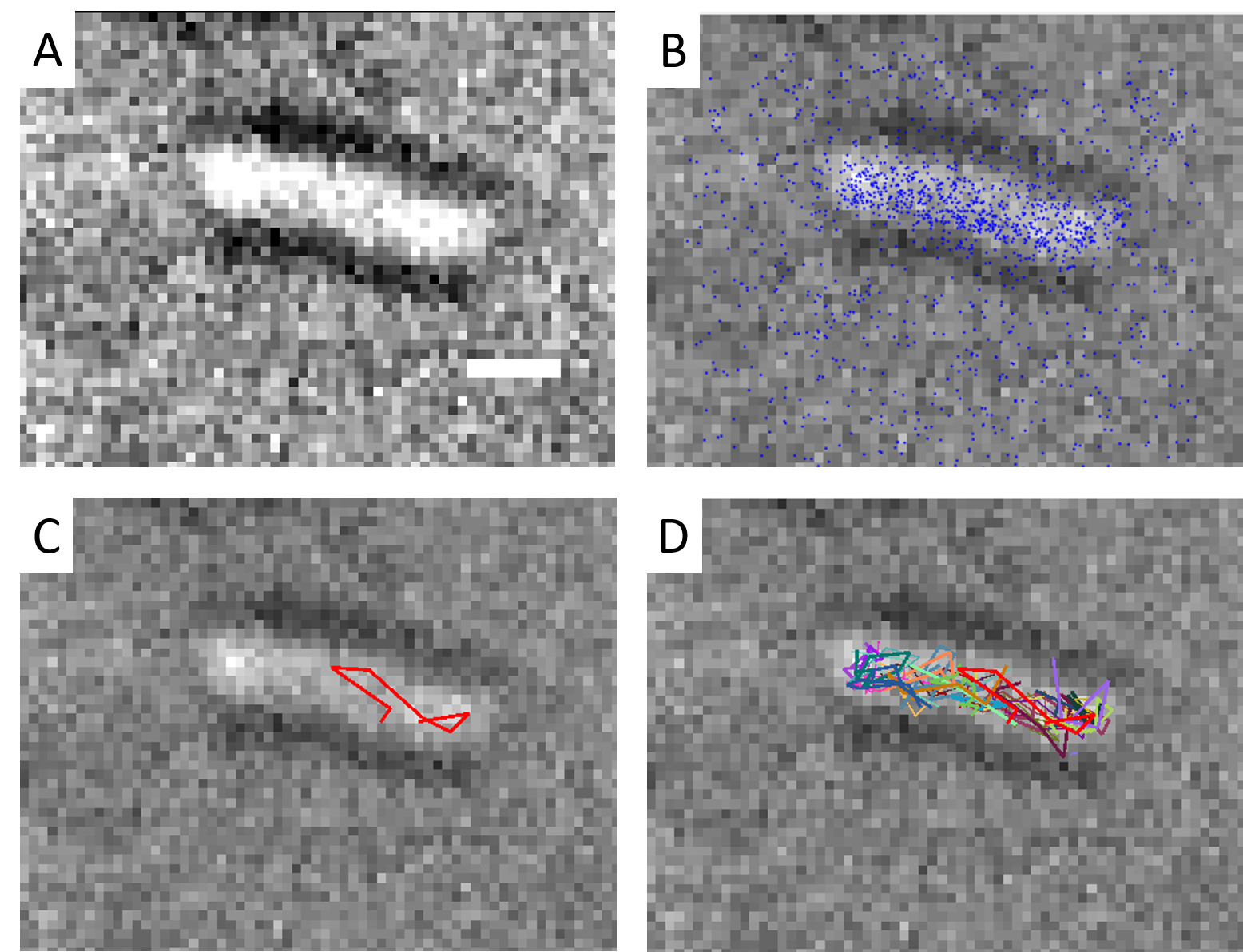

Supplement: FIG S3 [file mbio.03672-21-sf003.tif]
